# Emotional intelligence, perceived stress, and life satisfaction among university students: examining direct and moderating relationships

**DOI:** 10.3389/fpsyg.2026.1739364

**Published:** 2026-04-13

**Authors:** Manuel Gómez-López, Ignazio Leale, Antonio Martinez-Nicolas, David Manzano-Sánchez, Giuseppe Battaglia

**Affiliations:** 1 Department of Physical Activity and Sport, Faculty of Sport Sciences, University of Murcia, Murcia, Spain; 2 Sport and Exercise Research Unit, Department of Psychology, Educational Sciences and Human Movement, University of Palermo, Palermo, Italy; 3 Department of Physiology, Human Physiology Area, Sports Sciences Faculty, University of Murcia, Murcia, Spain; 4 Chronobiology Lab, Department of Physiology, College of Biology, Universidad de Murcia, Murcia, Spain; 5 Centro de Investigación Biomédica en Red de Fragilidad y Envejecimiento Saludable (CIBERFES), Madrid, Spain; 6 Department of Artistic, Musical and Dynamic Expression, Faculty of Education, University of Murcia, Murcia, Spain

**Keywords:** emotional intelligence, emotional regulation, life satisfaction, perceived stress, university students

## Abstract

University students are frequently exposed to academic and psychosocial stressors that may affect their psychological well-being. This study examined the relationships between perceived stress, emotional intelligence, and life satisfaction among university students. A cross-sectional design was conducted with 231 students from Spain and Italy enrolled in Physical Activity and Sport Sciences programs. Participants completed the Satisfaction with Life Scale (SWLS), the Perceived Stress Scale (PSS-14), and the Trait Meta-Mood Scale (TMMS). Results indicated that perceived stress was negatively associated with life satisfaction, whereas emotional intelligence dimensions were positively related to life satisfaction. However, emotional intelligence did not moderate the relationship between stress and life satisfaction. In addition, Spanish students reported lower stress and higher life satisfaction than Italian students. These findings suggest that emotional intelligence contributes to student well-being primarily through direct associations with life satisfaction rather than by buffering the effects of stress.

## 1 Introduction

The university stage generally encompasses the age range between 18 and 25 years. It is a unique period characterized by profound psychosocial, cognitive, and emotional changes. Arnett (2000) refers to this stage as emerging adulthood. During this period, university students experience changes in their life goals and interpersonal relationships. They also gain greater autonomy in decision-making, undergo constant exploration and reorganization of their identity, and progressively consolidate their personal and professional development.

In this context, life satisfaction (SWL) is an essential indicator of students’ psychological well-being, as it encompasses aspects related to emotional, social, and academic functioning (López-Guerra et al., 2025; Renshaw and Cohen, 2014). SWL can be defined as the cognitive dimension of subjective well-being, that is, the overall evaluation a person makes of the quality of their life according to their own criteria (Diener et al., 1985). It is positively related to the sense of control, the experience of pleasant emotions, and maintaining a clear life purpose (Martín-Talavera et al., 2024). In Diener et al.’s (1999) model of subjective well-being, two main components are distinguished: the affective one, based on the presence of positive emotions and the absence of negative ones; and the cognitive one, referring to life satisfaction. The latter is a stable component that reflects the consistency of a person’s general psychological adjustment.

Various studies have confirmed that SWL predicts mental health, psychological well-being, and adaptive capacity among university students when facing academic and personal stressors (Lee, 2024; Rogowska et al., 2021; Yıldırım et al., 2025). It should be noted that university students are in a vulnerable period, exposed to academic, social, and economic pressures (Pedrelli et al., 2015; Yu, 2024).

The literature has shown that students with higher levels of SWL tend to enjoy better mental health, show fewer symptoms of depression and anxiety, and achieve greater academic performance (Liu and Wang, 2024; Ooi et al., 2022). Conversely, when SWL is low, greater psychological fragility, risk behaviors, and difficulties adapting to academic and social environments are often observed (Bozkur and Güler, 2023; Olson et al., 2025; Wang et al., 2022). Recently, López-Guerra et al. (2025) confirmed that SWL scores correlated negatively with symptoms of depression and academic stress, and positively with emotional regulation strategies and resilience. Scientific evidence shows that SWL is positively related to emotional intelligence (EI), both in the general population and among university students, due to factors such as adequate self-perception and effective emotional regulation (Azpiazu et al., 2025; Escoda and Alegre, 2016; Fernández-Abascal and Martín-Díaz, 2019).

These findings highlight the importance of assessing life satisfaction as a key indicator of psychological well-being, mental health, and academic adjustment within the university context. In addition, cultural context may play an important role in shaping university students’ psychological adjustment and well-being. Previous research has shown that the transition to adulthood may follow different patterns across Mediterranean countries such as Spain and Italy, influenced by family structures, social support networks, and educational contexts (Pace et al., 2016). Considering these contextual differences may therefore contribute to a better understanding of students’ stress experiences and life satisfaction.

Moreover, many university students face the challenge of studying while also managing work or family responsibilities, which increases their mental and emotional load. Recent research, such as that by Simões de Almeida et al. (2025), indicates that factors like living away from the family home increase levels of depression and stress, significantly influencing subjective well-being. In addition, the current academic environment and the expectations of family and society impose demanding goals, excessive workloads, and promote self-demand, competitiveness, and constant comparison with others, factors that often foster academic stress, anxiety, and self-criticism (Córdova Olivera et al., 2023; Simões de Almeida et al., 2025). Alongside financial constraints and uncertainty about their professional future, these represent the main sources of pressure for university students.

Compared with other life stages, the university period represents a particularly sensitive stage in which symptoms of stress and other psychological difficulties may emerge, making students especially vulnerable to mental health problems (Barbayannis et al., 2022; Emmerton et al., 2024; Haruna et al., 2025; Pedrelli et al., 2015). Pérez-Jorge et al. (2025) highlight the progressive deterioration in the global psychological well-being of university students in recent years, especially after the COVID-19 pandemic.

As seen so far, within the university context, stress is associated with a lack of control and anxiety, and with academic exhaustion caused by overload and excessive demands, ultimately leading to reduced academic performance (Calatayud Mendoza et al., 2022). Lazarus and Folkman (1984) defined perceived stress as the way a person evaluates environmental demands and the resources available to cope with them. Thus, stress arises when academic demands exceed personal coping resources.

This perceived stress affects not only mental health but also physical and emotional well-being. It often manifests as a lack of control, anxiety, ruminative thoughts, irritability, emotional distress, and constant fatigue, all of which undermine well-being and SWL (Ansari et al., 2025; Bahl et al., 2023; Yildirim-Hamurcu and Terzioglu, 2022). For this reason, Folkman (2013) identified perceived stress as one of the main risk factors for students’ mental health.

However, several studies have shown that individuals with higher EI tend to experience lower levels of perceived stress (Ciarrochi et al., 2002; Extremera et al., 2009; Gey et al., 2025). This is due to emotional management, the ability to reinterpret difficult and negative situations in a more constructive way while maintaining emotional balance. The term emotional intelligence was defined by Salovey and Mayer (1990) as the ability to recognize, understand, and manage one’s own emotions and those of others. Later, Goleman (1996) expanded this concept by highlighting its importance in personal and social domains. Currently, the construct is understood to include three dimensions — attention, clarity, and repair (Fernández-Berrocal and Extremera, 2008a, 2008b) representing distinct processes: recognizing and attending to one’s emotions, understanding their meaning and nature, and regulating or transforming them in a balanced and constructive way.

The relationship between perceived stress and EI has been widely studied in recent years. Recent evidence indicates that EI can play a protective role, moderating the effect of stress on psychological well-being and life satisfaction and reducing its negative impact (Azpiazu et al., 2025; Extremera et al., 2009; Gilar-Corbi et al., 2024; Qiu et al., 2023; Yildirim-Hamurcu and Terzioglu, 2022). In particular, studies using the Trait Meta-Mood Scale (TMMS) have shown that the dimensions of emotional clarity and emotional repair are positively related to life satisfaction and can buffer the detrimental impact of perceived stress on well-being (Extremera and Fernández-Berrocal, 2005). In this regard, clarity and emotional repair have been linked to better academic adjustment, lower exhaustion, higher SWL and subjective well-being, and lower stress levels (Acebes-Sánchez et al., 2025; Fernández-Berrocal and Extremera, 2008a, 2008b).

It has been demonstrated that people with greater emotional skills tend to handle stress more effectively and evaluate their lives more positively (Azpiazu et al., 2025; Extremera et al., 2009). Gilar-Corbi et al. (2024) showed that EI enhances emotional self-regulation and self-efficacy — both essential components for university students. Conversely, paying excessive attention to one’s emotions can lead to rumination, overidentification, over-analysis, and distress, which tend to decrease subjective well-being (Extremera and Fernández-Berrocal, 2006; Fernández-Abascal and Martín-Díaz, 2019; Hodzic et al., 2016).

Promoting emotional competencies and providing proper training in EI and cognitive coping strategies allow university students to improve their emotional self-regulation and manage academic stress in a healthier and more adaptive manner (Gilar-Corbi et al., 2025; Khorasani et al., 2023; Martín-Talavera et al., 2024). All of this contributes to building a more solid and resilient psychological profile capable of withstanding external pressures (Hodzic et al., 2016; Cejudo et al., 2016).

Recent research conducted with Spanish university populations has further highlighted the relevance of emotional competencies in promoting subjective well-being. Studies carried out in higher education settings have examined the joint role of emotional intelligence and related socio-emotional competencies in the affective and cognitive components of subjective well-being, showing that emotional skills are positively associated with happiness and life satisfaction among university students (Cejudo et al., 2016; García-Martínez et al., 2023). Moreover, intervention programs that integrate emotional, positive psychology, or mindfulness-based components into regular university courses have reported improvements in students’ emotion regulation, psychological adjustment, and indicators of subjective well-being, including life satisfaction (Caballero-García and Sánchez Ruiz, 2021; Gilar-Corbí et al., 2018; Luján Henríquez and Martín Rodríguez, 2021). Together, these findings highlight the importance of developing emotional competencies within higher education contexts and support the need to further examine the role of emotional intelligence in students’ psychological well-being. In this context, students enrolled in Physical Activity and Sport Sciences programs represent a particularly relevant population within the field of sport psychology, as their academic training and lifestyle are closely related to sport participation, physical activity, and health promotion contexts.

Therefore, although few studies have simultaneously analyzed the three dimensions of EI as moderating variables between stress and SWL among university students, the literature supports a model in which perceived stress reduces SWL, though this effect is mitigated by EI, particularly through the dimensions of clarity and emotional repair. In this way, higher EI decreases vulnerability to stress and helps strengthen students’ overall well-being. Consequently, the central hypothesis of this theoretical framework proposes that perceived stress negatively influences SWL, and that EI (evaluated through attention, clarity, and emotional repair) moderates this relationship, acting as a protective or buffering factor against its negative effect.

**Figure 1:**
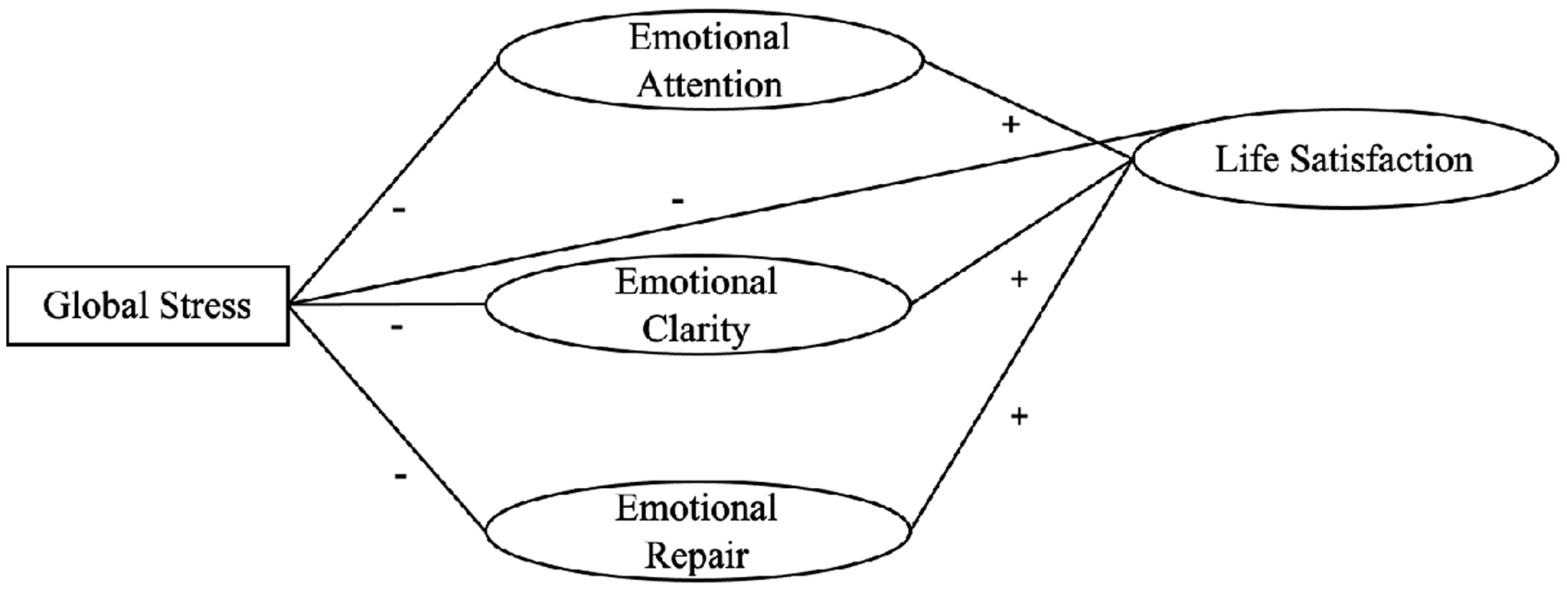
Study hypothesis.

From a theoretical perspective, the relationships among perceived stress, emotional intelligence, and life satisfaction can be understood within the framework of stress and coping models and emotional regulation theories. According to the transactional model of stress (Lazarus and Folkman, 1984), individuals’ appraisal of stressors and their available coping resources determine the psychological consequences of stressful situations. Emotional intelligence has been proposed as a relevant personal resource that facilitates emotional regulation, adaptive coping strategies, and more positive cognitive evaluations of life circumstances. Consequently, individuals with higher levels of emotional intelligence are expected to report lower levels of perceived stress and greater life satisfaction, as emotional competencies allow more effective management of emotionally demanding situations. Figure 1 presents the conceptual model guiding the present study, illustrating the proposed relationships between perceived stress, emotional intelligence dimensions (attention, clarity, and repair), and life satisfaction.

Based on the existing literature, the following hypotheses were proposed:

*H1*: Perceived stress will be negatively associated with life satisfaction among university students.

*H2*: The dimensions of emotional intelligence (emotional attention, emotional clarity, and emotional repair) will be positively associated with life satisfaction.

*H3*: Emotional intelligence (attention, clarity, and emotional repair) will moderate the relationship between perceived stress and life satisfaction, such that higher emotional intelligence will weaken the negative association between stress and life satisfaction.

*H4*: Differences in perceived stress, emotional intelligence, and life satisfaction may emerge according to contextual factors such as country and sex.

## 2 Method

### 2.1 Sample description

The present study employed a cross-sectional, observational design aimed at examining the relationships between perceived stress, emotional intelligence, and life satisfaction among university students. Data were collected through self-report questionnaires administered in an academic setting. Participants were recruited using a non-probabilistic convenience sampling strategy, as students enrolled in Physical Activity and Sport Sciences courses at participating universities were invited to voluntarily complete the questionnaire during class sessions.

A total of 236 participants were recruited (231 after the removal of outliers, as described in the statistical analysis section). Of the participants, 77.1% (178) were men and 22.9% (53) were women. Of the participants, 130 were from universities in Spain (56.3%) and 101 from Italy (43.7%). Additionally, 88.7% of the participants reported being engaged in sports activities. However, no detailed information regarding the type, frequency, or competitive level of these activities was collected, as sport participation was only considered as a contextual characteristic of the sample. Participants’ mean age was 21.15 years (SD = 3.72; range = 18–50). All were students enrolled in the Degree in Physical Education and Sport Sciences.

The final sample size (*N* = 231) was considered adequate for the statistical analyses performed, including correlation, MANOVA, and moderation analyses, and is comparable to sample sizes reported in previous studies examining emotional intelligence and well-being in university populations. The decision to focus on students enrolled in Physical Activity and Sport Sciences programs was intentional in order to ensure a relatively homogeneous academic context and to reduce variability associated with different academic disciplines. However, this characteristic should be considered when interpreting the generalisability of the findings.

The inclusion of participants from universities in Spain and Italy allowed for an exploratory comparison between two European university contexts with similar educational structures but potentially different cultural and social environments.

Participants were recruited from universities in Spain and Italy (two universities in total). Students were enrolled in different years of the Physical Activity and Sport Sciences degree programs.

### 2.2 Measures

The selected instruments were chosen because they are widely used and well-established measures for assessing perceived stress, emotional intelligence, and life satisfaction in university populations. In addition, validated Spanish and Italian versions of these scales are available, which makes them suitable for cross-cultural research in European student samples.

*Perceived Stress*. The Spanish (EEP; Remor and Carrobles, 2001; Remor, 2006) and Italian (Mondo et al., 2021) adapted versions of the original Perceived Stress Scale (PSS-14; Cohen et al., 1983) were used. This scale measures the degree to which, during the past month, individuals have felt upset or worried, or, on the contrary, confident in their ability to handle personal problems, through 14 items. Scores range from 0 to 56, with higher scores indicating greater perceived stress. The scale uses a five-point Likert response format ranging from 0 (Never) to 4 (Very often). The *α* value was 0.80.

*Perceived Emotional Intelligence*. The Spanish (Fernández-Berrocal et al., 2004) and Italian (Giromini et al., 2017) adapted versions of the original Trait Meta-Mood Scale (TMMS; Salovey et al., 1995) were used. This scale measures perceived emotional intelligence through 24 items distributed across three 8-item subscales that assess emotional attention, clarity of feelings, and emotional repair. The emotional attention subscale reflects the extent to which people notice and think about their feelings (e.g., “I pay a lot of attention to feelings”); the clarity of feelings subscale assesses the ability to understand one’s mood (e.g., “I am clear about my feelings”); and the emotional repair subscale evaluates the extent to which individuals regulate and manage their emotions (e.g., “When I’m sad, I think about all the pleasures of life”). The scale was preceded by the introductory phrase: “Below you will find some statements about your emotions and feelings…” Responses were recorded on a five-point Likert scale ranging from strongly disagree (1) to strongly agree (5). In the present study, internal consistency analyses yielded: emotional attention, α = 0.89; emotional clarity, α = 0.93; emotional repair, α = 0.88.

*Perceived Life Satisfaction*. The Spanish (Atienza et al., 2000) and Italian (Di Fabio and Busoni, 2009; Di Fabio and Palazzeschi, 2012) adapted versions of the original Satisfaction With Life Scale (SWLS; Diener et al., 1985) were used. This scale measures life satisfaction through five items grouped into a single factor. The items are presented as follows: (a) “In most ways my life is close to my ideal,” (b) “So far I have gotten the important things I want in life,” (c) “I am satisfied with my life,” (d) “If I could live my life over, I would do almost everything the same,” and (e) “The conditions of my life are excellent.” Responses were recorded on a seven-point Likert scale ranging from strongly disagree (1) to strongly agree (7). The total score ranges from 5 (low satisfaction) to 25 (high satisfaction). The alpha’s Cronbach value was 0.95.

All instruments were administered using previously validated Spanish and Italian versions to ensure linguistic and cultural equivalence across both samples. Internal consistency coefficients reported in this study correspond to the reliability obtained in the present sample.

### 2.3 Procedure

Data collection was carried out through an *ad hoc* questionnaire designed specifically for this study and hosted on the Google Forms® platform. The instrument included three validated scales, with questions written clearly and precisely to facilitate honest responses and obtain reliable information. The questionnaire was administered online during the months of January, February, and March 2025.

Before distribution, the teaching staff of the involved courses were contacted to explain the objectives of the research and request their collaboration, so that students could access the questionnaire during class hours, preferably in practical sessions, where participation was more accessible.

Participants were thoroughly informed about the purpose of the study, the voluntary nature of their participation, and the confidentiality of data processing. Although the questionnaire was administered during class sessions to facilitate access to participants, participation was entirely voluntary and students were informed that they could decline participation without any academic consequences. They were reminded that there were no right or wrong answers and were encouraged to respond sincerely and honestly. To minimize potential response bias, anonymity was reinforced and participants were assured that their responses would be used exclusively for research purposes. This procedure was intended to reduce social desirability and encourage honest responses. In addition, all participants signed an informed consent form guaranteeing the anonymity of their responses and compliance with current data protection regulations.

The process took place in a collective environment but with individual responses, under the supervision of the researcher, who provided the necessary instructions and addressed any doubts that arose. Completion of the questionnaire took approximately 20 min. The same administration procedure and instructions were used in both countries to ensure equivalence in data collection conditions across the Spanish and Italian samples. The questionnaires were completed in a classroom environment under the supervision of the researcher, ensuring similar environmental conditions and minimizing potential interruptions during completion. No compensation was offered for participation, and every effort was made to maintain an atmosphere of respect and calm throughout the process.

The study was conducted in accordance with the ethical principles of the Declaration of Helsinki (World Medical Association, 2024) and was approved by the Research Ethics Committee of the University of Murcia (ID: 4447/2023).

### 2.4 Statistical analysis

The statistical analyses were selected to examine the relationships among the study variables and to test the study hypotheses. Descriptive statistics and correlation analyses were first conducted to explore associations between perceived stress, emotional intelligence dimensions, and life satisfaction.

First, the dataset was screened for outliers using the Mahalanobis distance, leading to the exclusion of five atypical cases (*p* < 0.001). Subsequently, descriptive statistics (means, standard deviations, skewness, and kurtosis) were calculated for all study variables to assess univariate normality. Exact skewness and kurtosis values for all variables are reported in Table 1; all indices fell well within the acceptable range of ±1.96, following the criteria of Field (2017). In addition, assumptions of homogeneity of variances and absence of multicollinearity were examined and were considered acceptable for the statistical procedures applied. The internal consistency of each scale was examined using Cronbach’s alpha, showing adequate reliability across all measures. After verifying data distribution, Spearman’s correlations were performed due to slight deviations from normality in one of the variables. Multivariate analyses of variance (MANOVA) were performed to examine potential differences according to country and sex across the set of dependent variables simultaneously. This approach allows the assessment of group differences while controlling for Type I error inflation associated with multiple univariate comparisons. Although nonparametric procedures were also tested for comparison, both approaches yielded similar results; thus, MANOVA was retained for subsequent analyses.

**Table 1:** Descriptives and correlations results.

	Variables	M	SD	S	*K*	*α*	1	2	3	4	5
1	Global_Stress	1.70	0.56	0.115	−0.306	0.797	1	0.510**	−0.017	0.399**	0.490**
2	Life_Satisfaction	3.65	0.95	−0.395	−0.618	0.891		1	0.257**	0.456**	0.602**
3	Emotional_Attention	3.38	0.79	−063	−0.322	0.886			1	0.537**	0.353**
4	Emotional_Clarity	3.35	0.88	−118	−0.547	0.931				1	0.618**
5	Emotional_Repair	3.48	0.82	−126	−0.513	0.877					1

* *p* < 0.01; S, Skewness; K, Kurtosis.

Finally, a moderation analysis was conducted to examine whether emotional intelligence dimensions, specifically, emotional attention, emotional clarity, and emotional repair, moderated the relationship between perceived stress and life satisfaction. Because emotional intelligence was conceptualized as a multidimensional construct composed of attention, clarity, and repair, each dimension was analysed separately as a moderator in order to examine their potential differential effects on the relationship between perceived stress and life satisfaction. Each dimension of emotional intelligence was tested separately as a moderator using PROCESS Macro, Model 1 (Hayes, 2022) in IBM SPSS Statistics 25.0. Sex and country were included as covariates in the moderation models to rigorously control for demographic and cultural differences, as prior literature suggests these factors can significantly influence the baseline levels of perceived stress and life satisfaction (Pace et al., 2016). All continuous predictors were mean-centered before computing the interaction terms. All continuous predictors were mean-centered before computing the interaction terms. The analysis employed a bootstrapping procedure with 5.000 resamples to estimate bias-corrected 95% confidence intervals for conditional effects. A sensitivity power analysis indicated that the final sample size (*N* = 231) provides exceeding 80% statistical power (1–β > 0.80) to detect small-to-moderate effect sizes in regression-based moderation models, assuming an alpha level of 0.05. This confirms that the sample was highly adequate for the specific interaction analyses conducted.

## 3 Results

### 3.1 Descriptive analysis

Table 1 presents the descriptive statistics (mean and standard deviation), skewness (A) and kurtosis (C) indices, internal consistency (Cronbach’s *α*), and bivariate correlations for the study variables. The means indicate moderately low levels of perceived stress (M = 1.70, SD = 0.56) and medium-to-high levels of life satisfaction (M = 3.65, SD = 0.95). Regarding emotional intelligence, participants reported medium scores on emotional attention (M = 3.38, SD = 0.79), emotional clarity (M = 3.35, SD = 0.88), and emotional repair (M = 3.48, SD = 0.82). Internal consistency was adequate across all scales (α = 0.797–0.931), indicating high reliability of the measures. Regarding correlations, perceived stress (PSS_Global) was negatively and significantly correlated with life satisfaction (*r* = −0.510, *p* < 0.01), emotional clarity (*r* = −0.399, *p* < 0.01), and emotional repair (*r* = −0.490, *p* < 0.01). No significant association was found between perceived stress and emotional attention (*r* = −0.017, *p* > 0.05) and life satisfaction was positively and significantly associated with all emotional intelligence dimensions: attention (*r* = 0.257, *p* < 0.01), clarity (*r* = 0.456, *p* < 0.01), and repair (*r* = 0.602, *p* < 0.01). Furthermore, the emotional intelligence dimensions were positively correlated with each other, with coefficients ranging from *r* = 0.353 to *r* = 0.618 (*p* < 0.01).

### 3.2 Multivariate analysis

According to country (Table 2) The multivariate analysis of variance (MANOVA), evaluated using Wilks’ Lambda, indicated a significant overall effect on the combined dependent variables, Wilks’ Λ = 0.799, *F*(5, 225) = 11.33, *p* < 0.001 (Box’s value = 47.569; *f* = 3.096; *p* > 0.001), suggesting that country explains a meaningful portion of variance in the set of dependent variables. Follow-up univariate analyses revealed that Spanish participants reported lower perceived stress (M = 1.57, SD = 0.60) than Italian participants (M = 1.86, SD = 0.47), *F*(1, 229) = 15.66, *p* < 0.001, η^2^ = 0.064, and higher life satisfaction (M = 3.98, SD = 0.76 vs. M = 3.22, SD = 1.02), F(1, 229) = 41.74, *p* < 0.001, η^2^ = 0.154. Significant differences were also found in emotional clarity (*F* = 10.06, *p* < 0.01, η^2^ = 0.042) and emotional repair (*F* = 39.56, *p* < 0.001, η^2^ = 0.147), with Spanish students scoring higher on both dimensions. No significant differences were observed in emotional attention.

**Table 2:** Multivariate analysis according country.

Variables	Spain (*n* = 130) 56.3%		Italy (*n* = 101) 43.7%		*F*	*ŋ* ^2^
	M	SD	M	SD		
Global_stress	1.57	0.60	1.86	0.47	15.663**	0.064
Life_satisfaction	3.98	0.76	3.22	1.02	41.737**	0.154
Emotional_attention	3.41	0.83	3.34	0.74	0.440	0.002
Emotional_clarity	3.51	0.83	3.15	0.90	10.056**	0.042
Emotional_repair	3.76	0.82	3.13	0.67	39.563**	0.147
Wilk’s Λ					0.799	
*F* multivariate effect					11.328	

** *p* < 0.01; M, Mean; SD, Standard Deviation; ŋ^2^ = Partial eta Squared.

On the other hand, according sex (Table 3) the MANOVA testing the effect of sex on the combined variables showed a nonsignificant multivariate effect, Wilks’ Λ = 0.964, F(5, 225) = 1.70, *p* = 0.149 (Box value = 16.275; *f* = 1.045; *p* = 0.404), However, follow-up univariate analyses indicated a significant difference in emotional attention, with women scoring higher (M = 3.59, SD = 0.71) than men (M = 3.32, SD = 0.80), *F*(1, 230) = 5.04, *p* < 0.05, η^2^ = 0.022. No other variables, including perceived stress, life satisfaction, emotional clarity, or emotional repair, differed significantly by sex.

**Table 3:** Multivariate analysis according sex.

Variables	Women (*n* = 53) 22.9%		Men (*n* = 178) 77.1%		*F*	*ŋ* ^2^
	M	DT	M	DT		
Global_stress	1.81	0.59	1.66	0.55	2.820	0.012
Life_satisfaction	3.69	0.94	3.63	0.96	0.177	0.001
Emotional_attention	3.59	0.71	3.32	0.80	5.044*	0.022
Emotional_clarity	3.42	0.94	3.34	0.86	0.329	0.001
Emotional_repair	3.50	0.89	3.48	0.80	0.018	0.000
Global_stress					0.964	
Life_satisfaction					1.698	

** *p* < 0.01; M, Mean; SD, Standard Deviation; ŋ^2^ = Partial eta Squared.

### 3.3 Results of moderation analysis

The present analyses examined whether the three dimensions of emotional intelligence moderate the relationship between perceived stress and life satisfaction. Hierarchical regression analyses were conducted for each dimension, controlling for sex and country.

According to Table 4, perceived stress was negatively associated with life satisfaction (B = −0.612, *p* < 0.001), whereas emotional clarity was positively related to life satisfaction (B = 0.287, *p* < 0.001). The interaction between stress and clarity was not statistically significant (B = 0.140, *p* = 0.146), indicating that clarity did not significantly moderate the effect of stress on life satisfaction. The inclusion of the interaction term accounted for a small, non-significant increase in explained variance (ΔR^2^ = 0.006).

**Table 4:** Moderating role of emotional clarity in the relationship between stress and life satisfaction.

Predictor	B	SE	*t*	*p*	LLCI	ULCI
Constant	4.4757	0.1814	24.674	0.000	4.1183	4.8332
Stress	−0.6122	0.0990	−6.182	0.000	−0.8073	−0.4170
Emotional clarity	0.2874	0.0618	4.649	0.000	0.1656	0.4093
Stress × emotional clarity	0.1397	0.0958	1.458	0.146	−0.0491	0.3285
Sex	−0.1462	0.1180	−1.240	0.216	−0.3787	0.0862
Country	−0.4799	0.1029	−4.664	0.000	−0.6826	−0.2771

*R* = 0.6352. *R*^2^ = 0.4035. ΔR^2^ interaction = 0.0056. *F*-change = 2.125. *p* = 0.1463.

Regarding emotional attention, Table 5 shows that stress negatively predicted life satisfaction (B = −0.765, *p* < 0.001), and attention was positively related to life satisfaction (B = 0.298, *p* < 0.001). The interaction term was again non-significant (B = 0.020, *p* = 0.852), suggesting that attention does not significantly buffer or exacerbate the relationship between stress and life satisfaction. The change in *R*^2^ due to the interaction was negligible (ΔR^2^ = 0.0001).

**Table 5:** Moderating role of emotional attention in the relationship between stress and life satisfaction.

Predictor	B	SE	*t*	*p*	LLCI	ULCI
Constant	4.4651	0.1804	24.753	0.000	4.1096	4.8205
Stress	−0.7647	0.092	−8.315	0.000	−0.946	−0.5835
Emotional attention	0.2977	0.0632	4.709	0.000	0.1731	0.4222
Stress × attention	0.0199	0.1066	0.187	0.852	−0.1901	0.23
Sex	−0.5143	0.1027	−5.005	0.000	−0.7168	−0.3118
Country	−0.1041	0.1189	−0.875	0.383	−0.3384	0.13

*R* = 0.6324. *R^2^* = 0.3999. Δ*R*^2^ interaction = 0.0001. *F*-change = 0.035. *p* = 0.8517.

Finally, regarding emotional repair, Table 6 indicates that stress negatively predicted life satisfaction (B = −0.491, *p* < 0.001), while Repair of Emotion was positively associated with life satisfaction (B = 0.455, *p* < 0.001). The interaction between stress and Repair was not statistically significant (B = 0.128, *p* = 0.184), suggesting that Repair does not significantly moderate the impact of stress on life satisfaction. The interaction accounted for a small and non-significant increase in explained variance (ΔR^2^ = 0.004).

**Table 6:** Moderating role of emotional reparation in the relationship between stress and life satisfaction.

Predictor	B	SE	*t*	*p*	LLCI	ULCI
Constant	4.2791	0.1846	23.178	0.000	3.9153	4.6429
Stress	−0.4908	0.0981	−5.005	0.000	−0.684	−0.2975
Emotional reparation	0.455	0.0699	6.5120	0.000	0.3173	0.5927
Stress × reparation	0.1284	0.0963	1.334	0.184	−0.0613	0.3181
Sex	−0.1474	0.1131	−1.303	0.194	−0.3703	0.0756
Country	−0.3414	0.1033	−3.305	0.001	−0.545	−0.1378

*R* = 0.6745. *R*^2^ = 0.4549. Δ*R*^2^ interaction = 0.0043. *F*-change 1.779. *p* = 0.1836.

**Figure 2:**
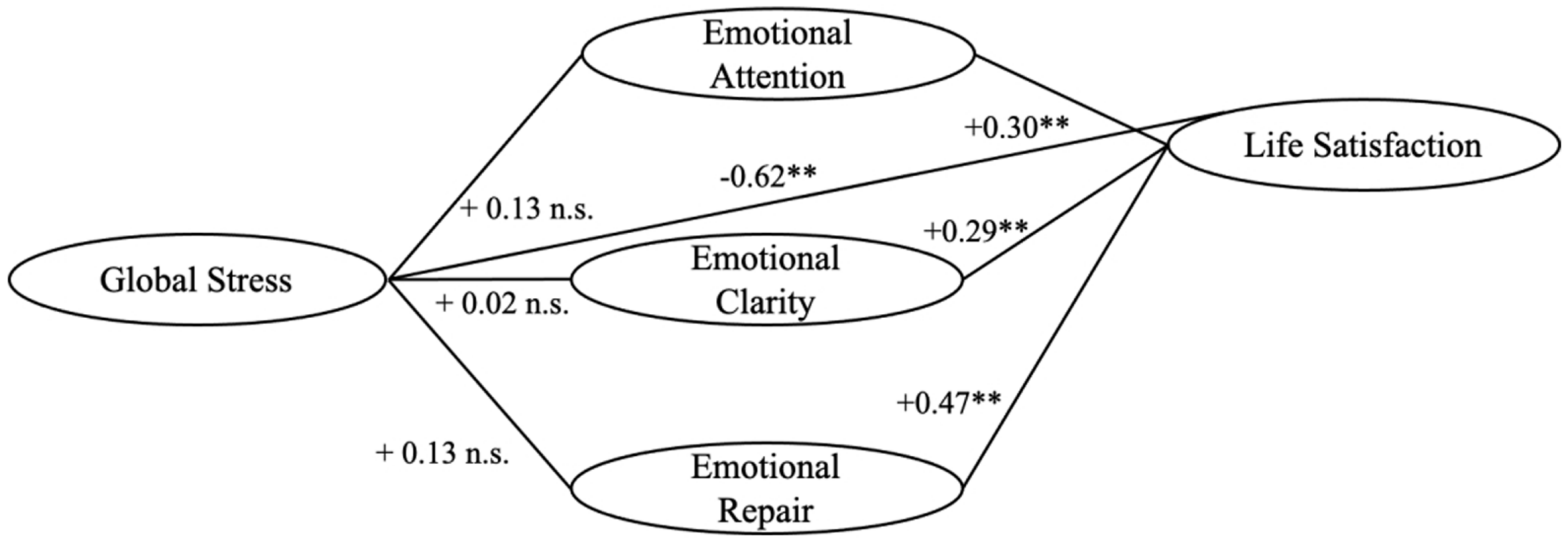
Moderating model of emotional intelligence dimensions on the relationship between global stress and life satisfaction. ** *p* < 0.01. ns, non-significant interaction. Models controlled for sex and country.

Finally, Figure 2 illustrates the direct and moderating effects of Global Stress and the three dimensions of Emotional Intelligence (Emotional Attention, Emotional Clarity, and Emotional Repair) on Life Satisfaction. As shown, Global Stress had a significant negative direct effect on Life Satisfaction (B = −0.62, *p* < 0.001). This value represents the averaged direct effect of Perceived Stress across the three separate moderation models (B = −0.61, −0.76, and −0.49, respectively). Figure 2 is intended as an integrated visual summary of the overall conceptual findings; exact coefficients and interaction terms for each separate dimension can be found in Tables 4–6. Each Emotional Intelligence dimension displayed a significant positive direct association with Life Satisfaction (B = 0.30, 0.29, and 0.46, respectively), indicating that higher levels of emotional competencies are related to greater life satisfaction. The interaction terms (Stress × Emotional Intelligence dimensions) were not significant, suggesting that Emotional Intelligence did not moderate the relationship between Stress and Life Satisfaction. Overall, the model highlights independent contributions of Stress and Emotional Intelligence to subjective well-being.

Overall, these results indicate that emotional intelligence dimensions were positively associated with life satisfaction, whereas perceived stress showed a negative association. However, none of the emotional intelligence dimensions significantly moderated the relationship between perceived stress and life satisfaction. These findings suggest that emotional intelligence is directly associated with life satisfaction rather than moderating the relationship between perceived stress and life satisfaction.

## 4 Discussion

This study examined the associations between perceived stress, emotional intelligence, and life satisfaction among university students in Italy and Spain, focusing on gender differences and cross-national comparisons. The results indicate that perceived stress is negatively associated with life satisfaction, while emotional intelligence shows a positive correlation with life satisfaction. However, none of the emotional intelligence dimensions (Emotional Attention, Emotional Clarity, and Emotional Repair) moderated the relationship between perceived stress and life satisfaction, suggesting that emotional skills may promote well-being through mechanisms independent of stress. This finding suggests that emotional intelligence may influence students’ well-being primarily through direct associations with life satisfaction rather than by buffering the negative effects of stress. In addition, the moderation models explained only minimal additional variance in life satisfaction (ΔR^2^ ranging from 0.0001 to 0.006), indicating that the interaction effects between perceived stress and emotional intelligence dimensions were negligible. It is also important to consider that moderation effects are typically more difficult to detect statistically than direct associations. Although the sample size was comparable to previous studies in this field, future research with larger samples may provide greater statistical power to examine potential interaction effects between perceived stress and emotional intelligence.

The absence of a significant moderation effect in the present study contrasts with some previous research suggesting that emotional intelligence may buffer the negative impact of stress on well-being. Several factors may explain this discrepancy. First, differences in sample characteristics may play a role, as the present study focused on students from a specific academic field (Physical Activity and Sport Sciences), whose levels of physical activity and coping resources may differ from those of the general university population. Second, emotional intelligence may operate more strongly as a direct psychological resource influencing life satisfaction rather than as a moderating factor between stress and well-being. Finally, contextual and cultural differences across university environments may also influence how emotional competencies interact with perceived stress.

From a theoretical perspective, these findings can be interpreted within the framework of Lazarus and Folkman’s transactional model of stress and coping, which emphasizes the role of cognitive appraisal and coping resources in determining the psychological consequences of stressful situations. Emotional intelligence may therefore function as a personal resource that facilitates more adaptive emotional regulation and coping strategies, contributing directly to higher life satisfaction even when it does not significantly modify the relationship between perceived stress and well-being.

These findings align with previous research showing that perceived stress negatively affects subjective well-being (Arslan et al., 2024), while emotional intelligence acts as a positive determinant of life satisfaction (Ruiz-Aranda et al., 2014). This suggests that emotional intelligence enhances life satisfaction primarily through improved emotional awareness and regulation, rather than by directly mitigating stress responses. This interpretation is also consistent with previous evidence indicating that emotional intelligence functions as a general resource for well-being rather than as a protective factor against stress (Malinauskas and Malinauskiene, 2020). Beyond individual differences, contextual and cultural factors also appeared to play an important role. The differences observed between Spanish and Italian students revealed that Spanish students reported lower levels of perceived stress, higher life satisfaction, and better scores in emotional clarity and repair. These findings may be attributed to cultural variations between the two countries, differences in the availability of social support, and diverse models of transition from adolescence to adulthood (Pace et al., 2016). Consistent with previous research, these results highlight the importance of considering cultural context when examining the emotional and social correlates of well-being (Pourmand et al., 2021; Chun et al., 2006).

Regarding gender differences, only emotional attention differed significantly between men and women, with women reporting higher levels of emotional attention. This finding is in line with prior evidence indicating greater emotional sensitivity and awareness among women (Vasilopoulos and Ellefson, 2021). Similarly, Delhom et al. (2024) found significant gender differences across all three dimensions of emotional intelligence in a sample of 853 cognitively healthy Spanish adults, with women scoring higher, particularly in emotional attention. These results are consistent not only with psychological research but also with neuroimaging evidence showing that women exhibit greater neural activation, especially in the left amygdala, in response to negative emotional stimuli, suggesting a higher emotional sensitivity and attention to emotional cues (Stevens and Hamann, 2012).

No significant gender differences were observed in life satisfaction or perceived stress. This contrasts with previous findings, such as those of Graves et al. (2021), who reported that women exhibited higher levels of perceived stress and used a wider range of coping strategies, including self-distraction, emotional support, instrumental support, and venting. The absence of gender differences in stress levels in the present study may reflect cultural or contextual variations in the university environments or the increasing normalization of stress experiences among both men and women in academic settings.

This study has some limitations. First, the cross-sectional design prevents causal inferences about the relationship among the variables. Second, the use of self-report measures may have introduced response bias. Third, as the sample consisted exclusively of university students, the generalizability of the findings to other populations is limited. In addition, the proportion of male participants was notably higher than that of female participants, which should be considered when interpreting the analyses related to gender and may limit the generalization of gender comparisons. Furthermore, although most participants reported being engaged in sports activities, detailed information about the type or frequency of physical activity was not collected. Future research should incorporate more precise measures of sport participation to better understand its potential influence on students’ well-being. Despite the limitations, the study also presents several notable strengths. It examines the relationships between perceived stress, emotional intelligence, including its specific dimensions, and life satisfaction, providing an integrated perspective on constructs that are often investigated separately. The inclusion of two distinct cultural contexts (Spain and Italy) further strengthens the study, enabling the exploration of cross-cultural variations. Finally, the methodological rigor, including the use of appropriate statistical analyses, supports the robustness and reliability of the findings.

Future research should further explore the mechanisms linking emotional intelligence, perceived stress, and subjective well-being using longitudinal and experimental designs. In particular, intervention studies aimed at developing emotional intelligence and socio-emotional competencies in university contexts could help determine whether improvements in emotional skills lead to reductions in perceived stress and increases in life satisfaction. Additionally, future studies could examine potential mediating mechanisms, such as coping strategies, resilience, or social support, that may explain how emotional competencies influence psychological well-being in university populations.

### 4.1 Practical implications

The results of the present study have important practical implications for promoting psychological well-being in the university context. First, they highlight the key role of emotional skills. Therefore, training programs aimed at developing emotional intelligence may represent an effective strategy to enhance student well-being. Such programs could include socio-emotional education, experiential workshops, and targeted training courses. Secondly, although physical activity was not directly analysed as a variable in the present study, the characteristics of the sample—composed mainly of students enrolled in Physical Activity and Sport Sciences programs, many of whom engage regularly in sport—suggest that promoting active lifestyles within university environments may represent a complementary strategy to enhance psychological well-being and life satisfaction. Previous research has shown that regular physical activity reduces perceived stress, improves mood, and strengthens emotional self-regulation (Cao et al., 2021; Mahindru et al., 2023; Vasilopoulos and Ellefson, 2021). Finally, mindfulness practices and adaptive coping techniques could help students manage academic and personal pressures more effectively.

Although several strategies may contribute to improving students’ well-being, previous research suggests that structured emotional intelligence training programs and interventions aimed at developing emotional regulation skills may represent particularly promising approaches in university settings. However, the implementation of such initiatives may face practical challenges, including limited institutional resources, the need for staff training, and potential variability in students’ engagement with socio-emotional programs. Therefore, future initiatives should consider the feasibility of integrating emotional competence development into existing university curricula and student support services in order to maximize their effectiveness and sustainability.

## 5 Conclusion

The present study contributes to a deeper understanding of the relationships between perceived stress, emotional intelligence, and life satisfaction among Italian and Spanish university students. The observed cross-cultural differences show that Spanish students report lower levels of stress and higher levels of well-being and emotional skills than Italian students. These findings underscore the importance of considering cultural factors in the study of psychological well-being. Gender differences were minimal, with women showing greater emotional attention but similar levels of stress compared to men. Overall, the results emphasize the importance of promoting emotional intelligence as a key component in interventions aimed at supporting mental health and well-being in university settings. Although emotional intelligence did not significantly moderate the relationship between perceived stress and life satisfaction, the results highlight its independent contribution to students’ well-being. These findings should be interpreted considering the methodological limitations of the study, particularly the cross-sectional design and the use of self-report measures. Nevertheless, the study provides relevant evidence supporting the promotion of emotional competencies in university contexts as a potential strategy to enhance students’ psychological well-being and life satisfaction. Future research should adopt longitudinal or experimental designs to clarify the causal relationships between emotional intelligence, stress, and life satisfaction, and to evaluate the effectiveness of integrated interventions that combine emotional and physical components of well-being across different cultural contexts.

## Data Availability

The raw data supporting the conclusions of this article will be made available by the authors, without undue reservation.
